# Spatial immune profiling of the colorectal tumor microenvironment predicts good outcome in stage II patients

**DOI:** 10.1038/s41746-020-0275-x

**Published:** 2020-05-15

**Authors:** Ines P. Nearchou, Bethany M. Gwyther, Elena C. T. Georgiakakis, Christos G. Gavriel, Kate Lillard, Yoshiki Kajiwara, Hideki Ueno, David J. Harrison, Peter D. Caie

**Affiliations:** 1 Quantitative and Digital Pathology, School of Medicine, University of St Andrews, St Andrews, KY16 9TF UK; 2 Indica Labs, Inc, 2469 Corrales Rd Bldg A-3, Corrales, NM 87048 USA; 3 Department of Surgery, National Defense Medical College, 3-2 Namiki, Tokorozawa, Saitama, 359-8513 Japan; 4 Lothian University Hospitals, Little France Crescent, Edinburgh, EH16 4SA UK

**Keywords:** Cancer microenvironment, Computational biology and bioinformatics

## Abstract

Cellular subpopulations within the colorectal tumor microenvironment (TME) include CD3^+^ and CD8^+^ lymphocytes, CD68^+^ and CD163^+^ macrophages, and tumor buds (TBs), all of which have known prognostic significance in stage II colorectal cancer. However, the prognostic relevance of their spatial interactions remains unknown. Here, by applying automated image analysis and machine learning approaches, we evaluate the prognostic significance of these cellular subpopulations and their spatial interactions. Resultant data, from a training cohort retrospectively collated from Edinburgh, UK hospitals (*n* = 113), were used to create a combinatorial prognostic model, which identified a subpopulation of patients who exhibit 100% survival over a 5-year follow-up period. The combinatorial model integrated lymphocytic infiltration, the number of lymphocytes within 50-μm proximity to TBs, and the CD68^+^/CD163^+^ macrophage ratio. This finding was confirmed on an independent validation cohort, which included patients treated in Japan and Scotland (*n* = 117). This work shows that by analyzing multiple cellular subpopulations from the complex TME, it is possible to identify patients for whom surgical resection alone may be curative.

## Introduction

Surgical resection remains the gold standard treatment for stage II colorectal cancer (CRC) patients^1^ and results in a 5- year overall survival of ~80% of the patients^2^. Adjuvant chemotherapy is only offered to patients classed as high-risk, based on features pertaining to the tumor itself, such as tumor differentiation and vascular or lymphatic invasion^3^. However, confidently identifying patients for whom surgical resection alone will be curative, may require a more complex analysis than analyzing the tumor cells alone. This is because the tumor microenvironment (TME) plays an important role in disease progression and thus prognosis^4^.

A TME that is enriched in T lymphocytes, has been consistently associated with better patient outcome in CRC^5–9^. Specifically, quantification of CD3^+^ and CD8^+^ T-cell densities in two discrete regions of the tumor has been shown to outperform current tumor risk factors, such as differentiation, venous emboli, and lymphatic invasion when predicting patient outcome^7^. Another key prognostic factor in CRC are tumor buds (TBs)^10,11^. TBs are small cancer clusters of up to four cells, which are predominantly identified within the invasive front of the tumor^12^. TBs represent the tumor’s aggressive potential and their presence has been repeatedly associated with worse prognosis in CRC^10,11,13^. In addition, the spatial relationship between T lymphocytes and TBs was shown to hold prognostic significance in CRC, where patients with high numbers of lymphocytes surrounding TBs demonstrate better stage II CRC prognosis^14^.

Macrophages, a major component of the cellular milieu within the CRC TME, also play a role in tumor progression^15^. Macrophages’ function include the stimulation of lymphocyte and other immune cells in order to respond to pathogens^16^. Within the tumor setting, macrophages can aid tumor progression, for example by promoting angiogenesis^15,17^ and extracellular remodeling^18^, as well as play an antitumorigenic role through the direct killing of cancer cells^19,20^ and the recruitment of cytotoxic lymphocytes^21^. A number of studies have further shown that cancer cell signaling can alter the macrophage’s metabolic structuring mechanisms, which can result to tumor progression and resistance to therapy^22^. The prognostic significance of macrophage infiltration in CRC, is not clear. Some studies have shown an association of high tumor macrophage density with improved survival^23–25^, whereas others associate it with a poor patient outcome and more aggressive phenotypes^26,27^. In addition, a study by Koelzer et al., demonstrated that frequent contact between TBs and CD68^+^ macrophages was present in tumors with adverse pathological findings, such as higher grade and lymph node metastasis^25^. Furthermore, in a study by Trumpi et al., it was found that co-culturing of macrophages with patient-derived colonospheres promoted tumor budding^27^.

This study employs automated image analysis and machine learning to quantify not only the densities of macrophages, TBs, and tumor infiltrating lymphocytes, but also their spatial inter-relationships in patient samples derived from Scotland and Japan. We further investigate their prognostic relevance in stage II CRC.

## Results

### Patient characteristics

Patients’ clinicopathological characteristics are shown in Table 1. This study included a training cohort of 113 stage II CRC patients of which 56 were male and 57 were female, and 87 were diagnosed with pT3 stage and 26 with pT4 stage disease. Of these patients, seven rectal cancer patients received neo-adjuvant treatment and two colon cancer patients, who were positive for extramural lymphovascular invasion (EMLVI), received adjuvant treatment. The validation cohort included 117 stage II CRC patients of which 75 were male and 42 were female. One hundred patients in this cohort were diagnosed with pT3 stage and 17 were of pT4 stage disease. Four rectal cancer patients received neo-adjuvant therapy and 12 CRC patients underwent adjuvant therapy (only one of whom had confirmed EMLVI).

**Table 1:** Univariate Cox regression analysis for clinicopathological data, SIOI, and SIOI components for the training and validation cohort.

Features	Training cohort (*n* = 113)			Validation cohort (*n* = 117)		
	Freq (%)	HR (95% CI)	*P*	Freq (%)	HR (95% CI)	*P*
Clinicopathological						
Age		1.447 (0.771–2.715)	0.250		1.499 (0.921–2.440)	0.103
≤70	45 (39.8)			63 (53.8)		
71–79	32 28.3)			32 (27.4)		
≥80	36 (31.9)			22 (18.8)		
Gender		0.829 (0.301–2.287)	0.717		0.635 (0.252–1.600)	0.336
Male	56 (49.6)			75 (64.1)		
Female	57 (50.4)			42 (35.9)		
pT stage		**4.081 (1.461–11.390)**	**0.007**		**3.124 (1.293–7.548)**	**0.011**
pT3	87 (77.0)			100 (85.5)		
pT4	26 (23.0)			17 (14.5)		
Tumor site		0.666 (0.340–1.304)	0.236		1.461 (0.836–2.553)	0.183
Left	38 (33.6)			31 (26.5)		
Right	42 (37.2)			42 (35.9)		
Rectal	33 (29.2)			44 (37.6)		
Differentiation		2.072 (1.000–4.293)	0.050		1.008 (0.712–1.426)	0.965
Moderate	91 (80.5)			34 (29.1)		
Poor	19 (16.8)			14 (12.0)		
Well	3 (2.7)			68 (58.1)		
N/A	0			1(0.9)		
EMLVI		0.347 (0.118–1.015)	0.053		1.112 (0.729–1.697)	0.622
Yes	18 (15.9)			3 (2.6)		
No	95 (84.1)			34 (29.1)		
N/A	0			80 (68.4)		
Tumor Type		<0.001 (0–Inf)	0.998		1.632 (0.801–3.327)	0.178
Adenocarcinoma	104 (92.0)			100 (85.5)		
Mucinous	5 (4.4)			13 (11.1)		
Mixed	4 (3.5)			3 (2.6)		
N/A	0			1 (0.9)		
DSD—-full follow-up		NA			NA	
Yes	15 (13.3)			24 (20.5)		
No	98 (86.7)			93 (79.5)		
DSD—5-year follow-up		NA			NA	
Yes	9 (8.0)			17 (14.5)		
No	104 (92.0)			100 (85.5)		
Image analysis						
SIOI		**6.119 (2.661–14.07)**	**<0.001**		**1.960 (1.310–2.932)**	**0.001**
+/+/+	62 (54.9)			19 (16.2)		
+/+/−	30 (25.6)			47 (40.2)		
+/−/− or −/−/−	21 (18.6)			51 (43.6)		
CD3^+^ in IMCT		**9.803 (3.105–30.950)**	**<0.001**		**4.218 (1.779–10.000)**	**0.001**
Low	29 (25.7)			47 (40.2)		
High	84 (74.3)			70 (59.8)		
CD3^+^CD8^+^ 0–50-μm TB		**9.420 (2.996–29.620)**	**<0.001**		**2.617 (1.144–5.990)**	**0.023**
Low	27 (23.9)			47 (40.2)		
High	86 (76.1)			70 (59.8)		
CD68^+^/CD163^+^ in CT		**4.287 (1.550–11.860)**	**0.005**		1.317 (0.557–3.110)	0.531
Low	90 (79.6)			42 (35.9)		
High	23 (20.4)			75 (64.1)		

Significant features (*P* < 0.05) are shown in bold.

*Freq*. frequency, *DSD* disease-specific death.

### The association between macrophage density, lymphocytic density, and tumor budding

**Fig. 1:**
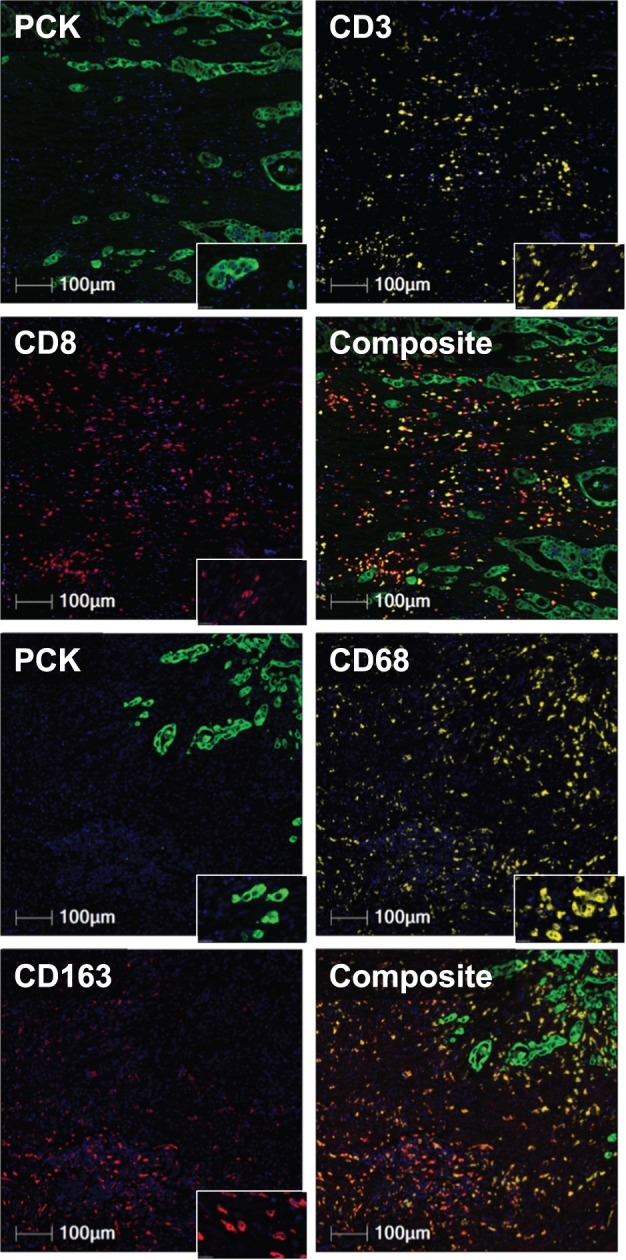
Multiplex immunofluorescence of the tumor cells, tumor infiltrating lymphocytes, and macrophages. Tumor cells are shown in green, CD3^+^ and CD68^+^ cells in yellow, CD8^+^ and CD163^+^ in red. Composite images for both slides are shown.

**Fig. 2:**
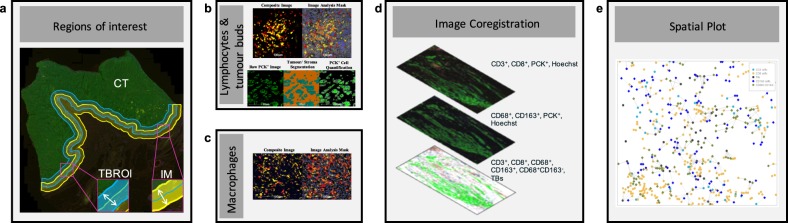
Automated image analysis workflow. **a** Regions of interest for quantification of features. The tumor core is shown in green (CT), the tumor bud (TB) region of interest in blue (TBROI), and the invasive margin in yellow (IM). **b** Composite image: CD3^+^ cells in yellow and CD8^+^ cells in red, image analysis mask: classification of lymphocytes, CD3^+^ cells in yellow, CD8^+^ cells in red and their colocalization (CD3^+^CD8^+^), based on image analysis thresholds, in orange, raw PCK^+^ image: pancytokeratin^+^ (PCK^+^) cells (epithelial cells) in green, tumor/stroma segmentation: tumor regions in turquoise and stroma regions in orange, PCK^+^ cell quantification: epithelial cell quantification within the tumor areas. **c** Composite image: CD68^+^ cells in yellow and CD163^+^ cells in red, image analysis mask: classification of macrophages, CD68^+^ cells in yellow, CD163^+^ cells in red and their colocalization in orange. **d** automatic image coregistration. **e** Spatial analysis for lymphocytes (CD3^+^ cells in light blue circles, CD8^+^ cells in orange circles), macrophages (CD68^+^CD163^−^ cells in green rhombus and CD163^+^ cells in blue rhombus) and TBs (gray circles). Proximity lines are shown for macrophages within 50-µm of TBs or lymphocytes.

**Fig. 3:**
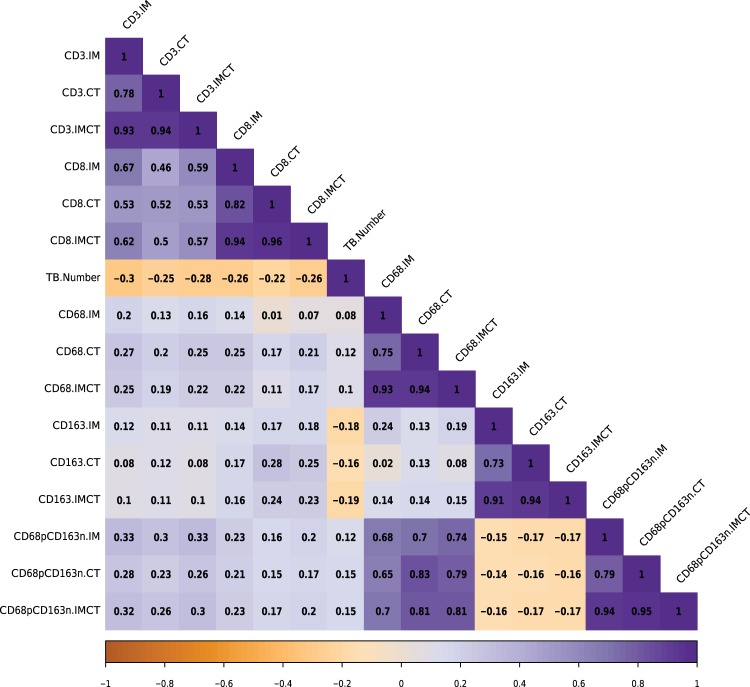
Spearman correlation matrix for macrophages, lymphocytes, and TBs. A correlation coefficient is shown for each relationship. A greater than 0 coefficient is shown in purple and indicates a positive association, a less than 0 coefficient is shown in orange and indicates a negative association.

Multiplex immunofluorescence was performed on two sequential CRC tissue sections for the visualization of CD3^+^, CD8^+^, CD68^+^, CD163^+^, CD68^+^CD163^−^, and pancytokeratin (PCK)^+^ cells (Fig. 1). Image analysis automatically quantified CD68^+^, CD163^+^, and CD68^+^CD163^−^ macrophage densities and CD3^+^ and CD8^+^ cells within the invasive margin (IM), tumor core (CT), and both IM and CT areas (IMCT) as well as TBs within the TB region of interest (TBROI, Fig. 2). Spearman correlation was used to evaluate their relationships and the resultant r coefficients are shown in Fig. 3. There was a weak positive correlation between TBs and CD68^+^ (r = 0.12) and TBs and CD68^+^CD163^-^ (r = 0.15) macrophage subpopulations in the CT. CD68^+^ density (IM, CT, IMCT) and CD163^+^ (IM) were weakly associated with CD3^+^ density in the IM (r = 0.20, r = 0.27, r = 0.25, and r = 0.12, respectively). CD68^+^ density in the CT was weakly correlated with CD3^+^ density in the IMCT (r = 0.25) as well as CD8^+^ density in the IM (r = 0.25). CD163^+^ density (IM, CT, IMCT) was weakly associated with CD8^+^ density in the IM (r = 0.14, r = 0.17, and r = 0.16, respectively), CT (r = 0.17, r = 0.28, and r = 0.24, respectively), and IMCT (r = 0.18, r = 0.25, and r = 0.23, respectively). CD68^+^CD163^-^ density (IM, CT, IMCT) was correlated with both CD3^+^ in the IM (r = 0.33, r = 0.28, and r = 0.32, respectively) and IMCT (r = 0.33, r = 0.26, and r = 0.30, respectively). TBs were inversely correlated with CD3^+^ density in the IM (r = −0.30), CT (r = −0.25), and IMCT (r = −0.28). TBs were also inversely correlated with CD8^+^ density in the IM (r = −0.26), CT (r = −0.22), and IMCT (r = −0.26). The *P*-values of these associations are listed in Supplementary Table 1.

### Prognostic model development

The number of features reported in this study was reduced to eliminate any features that were not significantly associated with prognosis. To do this, the 69 image analysis features, together with seven features from the clinicopathological report of the training cohort, were input into a Cox proportional hazard regression with the least absolute shrinkage and selection operator (LASSO) penalty. Results reported 11 significant features (Table 2). These 11 features were subsequently the input to a random forest analysis, which ranked them by their mean decrease Gini. Features with mean decrease Gini of greater than 3 were then selected and were binarized based on the training cohort’s survival data. In an iterative process, the least significant feature was then removed until its removal negatively affected the model’s prognostic value. This led to the development of a new prognostic system, termed the “Spatial Immuno-Oncology Index” (SIOI). The SIOI comprised of the following three image analysis features: the average CD3^+^ density in the IMCT, the average number of lymphocytes within 50-μm of TB and the CD68^+^/CD163^+^ ratio in the CT. The optimal cutoff points of these features are shown in Supplementary Table 2. Patients with a co-occurrence of high density of CD3^+^ cells in IMCT, high-average number of CD3^+^CD8^+^ cells within 50-μm of TB and low CD68^+^/CD163^+^ ratio in the CT were grouped in the “+/+/+” category. Patients presenting only two of these features were classed as “+/+/−” whereas patients with one or none of these features were classed as “+/−/− or −/−/−”.

**Table 2:** LASSO penalized Cox regression and Random Forest Gini coefficients for the significant features.

Features	Coefficients	
	LASSO	Mean decrease Gini
CD3^+^ in IMCT	−0.00057	4.28147
CD3^+^CD8^+^ within 0–50-μm of TB	−0.10314	3.90178
CD68^+^/CD163^+^ in CT	0.12435	3.61488
CD68^+^CD163^-^ in CT	0.00150	3.48619
CD8^+^ in CT	−0.00007	3.07934
TB Number	0.00008	2.68648
CD163^+^ within 0–50-μm of CD8^+^	−0.00034	2.43362
pT	0.89074	0.65122
EMLVI	−0.32011	0.60136
Age	0.38133	0.55309
Differentiation	0.61914	0.50729

### Survival analysis

**Fig. 4:**
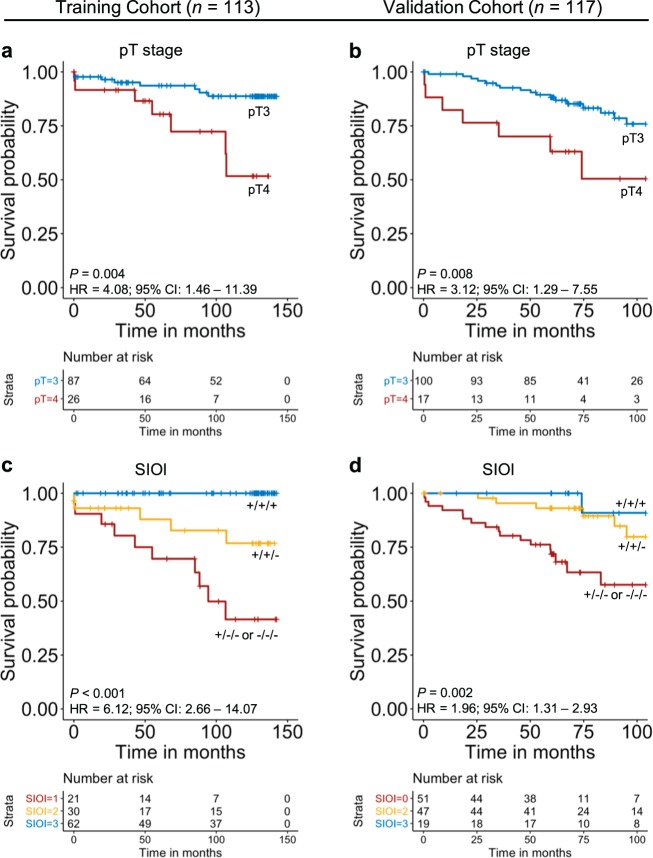
KM survival analysis for pT stage and SIOI for training cohort (11.8-year follow-up) and validation cohort (8.6-year follow-up). **a** pT stage for training cohort. **b** pT stage for validation cohort. **c** SIOI for training cohort. **d** SIOI for validation cohort. The “+/+/+” category represents the group of patients who have CD3^+^ density in the IMCT above the cutoff point (389.6 cells/mm^2^), average CD3^+^CD8^+^ number within 0–50-μm of TB above the cutoff point (4.1) and CD68^+^ /CD163^+^ ratio in the CT below the cutoff point (1.096); “+/+/−” group represents patients who are positive for only two of these features and “+/−/− or −/−/−” represents the group of patients who have only 1 or none of the above features. The HR is calculated using univariate Cox regression.

Univariate Cox regression was applied to the clinicopathological data of the training cohort to assess their prognostic significance. Only pT stage was significantly associated with disease-specific survival over the full follow-up period of 11.8 years (hazard ratio (HR) = 4.081; 95% CI, 1.461–11.390; *P* = 0.007, Table 1). Kaplan–Meier (KM) survival analysis (Fig. 4) was further performed and patients of pT4 stage had significantly lower disease-specific survival rate over 11.8 years follow-up (51.7%) in comparison to the patients with pT3 stage (88.8%; Fig. 4a). The independent prognostic value of the 11 features selected by Cox regression with LASSO regularization was also investigated using multivariate Cox regression and results are shown in Supplementary Table 3. To assess the prognostic significance of the SIOI, Cox regression and KM survival analysis were applied. When KM survival analysis was performed, “+/+/+” patients were shown to confer significantly better survival outcome (100% survival rate) when compared to the “+/+/−” patients (76.8% survival rate) or the “+/−/− or −/−/−” patients (41.5% survival rate, Fig. 4c). By applying a univariate Cox regression, the prognostic value of SIOI was again found to be highly significant (HR = 6.119; 95% CI, 2.661–14.069; *P* < 0.001, Table 1). Patients who received adjuvant treatment were then excluded from the cohort and univariate Cox regression was reapplied to assess SIOI’s prognostic value. Results showed that the prognostic significance of SIOI did not change significantly (HR = 6.407; 95% CI, 2.784–14.740; *P* < 0.001). Cox regression was also applied to the model’s individual components; CD3^+^ cells in IMCT (HR = 9.803; 95% CI, 3.105–30.950; *P* < 0.001), average number of CD3^+^CD8^+^ cells with 50-μm of TB (HR = 9.420; 95% CI, 2.996–29.620; *P* < 0.001) and CD68^+^/CD163^+^ ratio in CT (HR = 4.287; 95% CI, 1.550–11.860; *P* = 0.005, Table 1). When multivariate forward stepwise Cox regression was applied using SIOI, its composite parts and pT stage, SIOI was shown to be the only image-based variable to be prognostically significant (HR = 6.119; 95% CI, 2.661–14.069; *P* < 0.001), followed by pT stage (HR = 3.531; 95% CI, 1.260–9.893; *P* = 0.016, Supplementary Table 4). We then created a 2 category SIOI where “+/+/+” patients (low-risk) were compared to “+/+/−, +/−/−, −/−/−” (high-risk) patients. Results showed that the high-risk group included all 15 disease-specific deaths of the cohort therefore meaning that no deaths occurred within the patient group that predicated good prognosis. In comparison, when categorizing the patients based on pT stage, the pT4 group included only seven out of the 15 events (Supplementary Fig. 1).

**Fig. 5:**
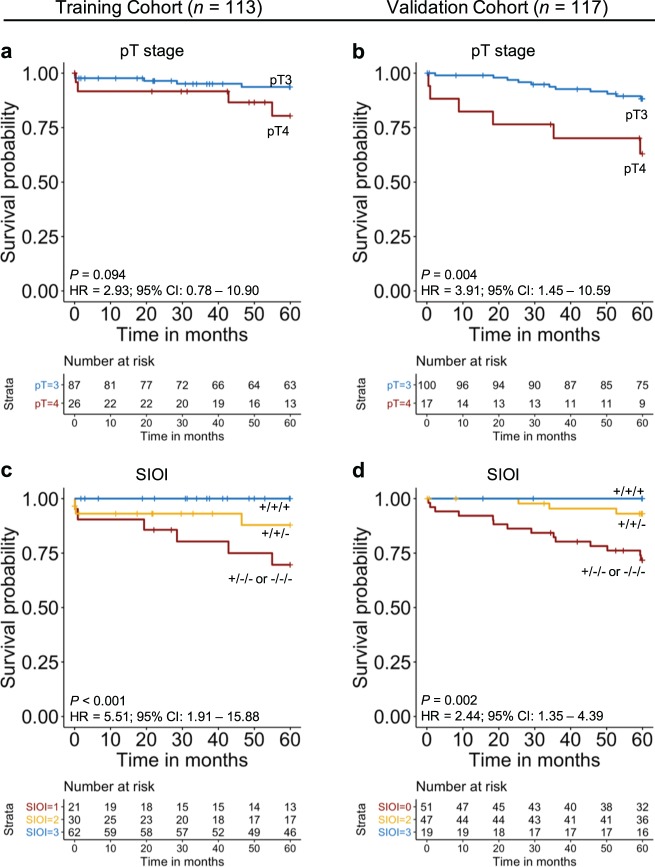
KM survival analysis for pT stage and SIOI for training cohort (5-year follow-up) and validation cohort (5-year follow-up). **a** pT stage for training cohort. **b** pT stage for validation cohort. **c** SIOI for training cohort. **d** SIOI for validation cohort. The “+/+/+” category represents the group of patients who have CD3^+^ density in the IMCT above the cutoff point (389.6 cells/mm^2^), average CD3^+^CD8^+^ number within 0–50-μm of TB above the cutoff point (4.1) and CD68^+^/CD163^+^ ratio in the CT below the cutoff point (1.096); “+/+/−” group represents patients who are positive for only two of these features and “+/−/− or −/−/−” represents the group of patients who have only 1 or none of the above features. The HR is calculated using univariate Cox regression.

The prognostic significance of SIOI was re-examined using 5-year follow-up data. KM survival analysis (Fig. 5) revealed that “+/+/+” patients conferred significantly better survival outcome (100% survival rate) when compared to patients of the “+/+/−” group (87.9% survival rate) or the “+/−/− or −/−/−” group (69.6% survival rate, Fig. 5c). Univariate Cox regression results also showed that SIOI was prognostically significant (HR = 5.508; 95% CI, 1.910–15.880; *P* = 0.002). SIOI was also the sole significant feature to predict disease-specific death on 5-year prognosis (HR = 5.508; 95% CI, 1.910–15.884; *P* = 0.002) when entered into a multivariate forward stepwise Cox regression model together with its composite parts and pT stage (Supplementary Table 5). When the 2 category SIOI was examined, results showed that the high-risk group included all seven disease-specific deaths for the 5-year follow-up (Supplementary Fig. 1c). In comparison, when categorizing the patients based on pT stage, the pT4 group included only four out of the seven events for the 5-year follow-up period. pT stage was not found to be significant when applying a univariate Cox regression using 5-year follow-up (HR = 2.925; 95% CI, 0.784–10.900; *P* = 0.110) or KM survival analysis (Fig. 5a).

### Validation of SIOI

SIOI was then assessed on an independent validation cohort (*n* = 117), which included 56 patients treated in Scotland and 61 patients treated in Japan. The training cohort cutoff points, calculated for each of the SIOI’s composite parameter, were applied to the validation cohort. SIOI was shown to be prognostically significant when assessed by univariate Cox regression using full follow-up (HR = 1.960; 95% CI, 1.310–2.932; *P* = 0.001, Table 1). Exclusion of patients who received adjuvant treatment did not alter the prognostic value of SIOI (HR = 1.958; 95% CI, 1.274–3.010; *P* = 0.002). The SIOI was also found to be prognostically significant using 5-year follow-up (HR = 2.440; 95% CI, 1.350–4.390; *P* = 0.003). The results from the KM survival analysis also showed that SIOI was significantly associated with patient survival (8.6-year: *P* = 0.002, Fig. 4d and 5-year: *P* = 0.002, Fig. 5d). For 8.6-year follow-up, only one disease-specific death was included in the low-risk category (“+/+/+”) whereas for 5-year follow-up all patients within the group survived. When testing the SIOI only on the Scottish subcohort of the validation cohort, one event was included in the low-risk group (“+/+/+”, Supplementary Fig. 2a) whereas when assessing it only on the Japanese subcohort, no events were observed in the low-risk group (Supplementary Fig. 2b) for the full follow-up period. pT stage was shown to be prognostically significant in the validation cohort when assessed by univariate Cox regression (8.6-year follow-up: HR = 3.124; 95% CI, 1.293–7.548; *P* = 0.011 and 5-year follow-up: HR = 3.912; 95% CI, 1.445–10.590; *P* = 0.007) or KM survival analysis (8.6-year: *P* = 0.008, Fig. 4b; 5-year: *P* = 0.004, Fig. 5b). When comparing the 2-tier SIOI to pT stage using 8.6-year follow-up, 16 out of the 17 disease-specific deaths were captured within the high-risk group of SIOI (“+/+/−, +/−/−, and −/−/−”, Supplementary Fig. 1b) whereas only six of these were captured by the pT4 stage group. Using 5-year follow-up, all 17 disease-specific deaths were included in the high-risk group whereas pT4 stage included only six out of the 17 events (Supplementary Fig. 1d).

## Discussion

The TME, which consists of several interacting cellular subpopulations including immune cells, is an influencing factor in tumor progression^28^. Studying multiple features of the TME, and their interactions, may provide a more accurate and personalized patient prognosis than by reporting single elements in isolation. In this study, we assess the prognostic significance of macrophage subpopulation densities and their ratios (CD68^+^, CD163^+^, and CD68^+^CD163^−^ expressing cells) within different regions of the tumor (IM, CT, IMCT) using automated image analysis. Furthermore, we assess the spatial relationships between macrophages, TBs and tumor infiltrating lymphocytes to further our understanding of their role in patient survival. Machine learning led to the development of a highly significant prognostic index. This index (SIOI) consisted of CD3^+^ infiltration densities in the IMCT, the average number of CD3^+^ and CD8^+^ cells within 50-μm proximity to TBs and a CD68^+^/CD163^+^ cellular ratio in the CT. When applying the SIOI to a training cohort of patients, it stratified a subpopulation of stage II CRC patients who experienced a 100% survival rate over both 5- and 11.8-year follow-up. This result was validated in an independent unseen patient cohort, which consisted of patients from Scotland and Japan. SIOI stratified a patient population in which 100% of patients survived 5-year follow-up and in which 95% survived 8.6-year follow-up.

Tumor-associated macrophages are key players within the TME and their function differs among distinct TMEs^15^. In CRC, the role of macrophages is controversial with a number of studies reporting their presence as a favorable prognostic factor^23–25^ and others demonstrating their association with adverse prognosis^26,27^. In this study, we found that a low CD68^+^/CD163^+^ cellular ratio in the CT was significantly associated with improved disease-specific survival. This suggests that rather than the overall macrophage densities being a significant factor, it is the proportion of the CD163^+^ subpopulation of macrophages that confers prognostic significance. Indeed, in previous work by Feng et al.^29^, CD206^+^/CD68^+^ ratio was shown to be a better prognostic biomarker than CD68^+^ or CD206^+^ cell density for disease-free survival and overall survival in stage II colon cancer. Results from this study also showed that a high CD206^+^/CD68^+^ ratio was correlated with poor disease-specific survival, which is in disagreement with our results. However, in that study, macrophage quantification was performed on stromal regions of tissue microarray cores, whereas in our study we assess the macrophage infiltration across whole-slide images to examine their different spatial distribution within the heterotypic and heterogeneous TME. In fact, in a study by Yang et al.^30^, the CD163^+^/CD68^+^ ratio was shown to be significantly associated with relapse free survival and overall survival in CRC only when assessed at the tumor front, but not in the CT. This therefore highlights that the clinicopathological implication of macrophages may vary according to their distinct location within the tumor, as also demonstrated in this study. Unlike macrophage density, the prognostic significance of tumor infiltrating lymphocytes in CRC is well established, with multiple studies reporting high lymphocytic infiltration to be associated with better patient survival outcome^7,8,14,31^. In line with previous results, we have shown that patients with high CD3^+^ infiltration densities in the IMCT confer better survival outcome than patients with low-lymphocytic infiltration.

The epithelial to mesenchymal transition (EMT), in which cells lose their epithelial properties such as cell–cell adhesion while gaining mesenchymal characteristics such as the ability to migrate, plays a critical role in tumor invasion and metastasis^32^. Previous studies have shown that macrophages have pro-tumorigenic properties by stimulating cell migration and EMT^33,34^. Additionally, TBs are regarded to have undergone EMT^35^ as their molecular characterization has previously revealed the gain of mesenchymal-like properties^36^. When this study assessed the relationship between macrophage densities and TB number, a weak positive correlation was observed. These results may suggest that tumor budding and hence tumor invasion might be driven through a mechanism relating to the macrophage function within the TME. A study by Trumpi et al.^27^, has reported that co-culture of macrophages with patient-derived colonospheres aided tumor-budding formation. An alternative possibility, might be that macrophages are recruited to high budding tumors for the destruction of these tumor cells, therefore acting in an antitumorigenic fashion. In fact, in a study by Li et al.^37^, patients with both high number of TBs and increased CD68^+^ macrophage infiltration conferred a favorable survival outcome, which was similar to patients with low tumor budding. Consistent with results by Li et al.^37^, we also observed a positive, although weak, association between lymphocytic and macrophage densities within the TME. This could suggest that macrophages act in an antitumorigenic fashion by inducing a T-cell lymphocytic response for the elimination of these aggressive tumor subpopulations. A weak negative association was observed between TBs and lymphocytic densities, which concur well with previous findings^38^. Future studies though would need to be conducted to confirm these observations as well as investigate and establish the molecular mechanisms behind tumor budding and their association with lymphocytic and macrophage density within the TME.

All image analysis features together with the data from the clinicopathological report were assessed for prognostic significance. In order to identify the most significant prognostic factors, a workflow consisting of Cox regression with LASSO regularization and random forest analysis was employed. These methods were selected in order to overcome any issues relating to highly correlated features as well those arising from complex, high dimensional data. Ten-fold cross validation and out-of-bag error were applied whilst running the Cox regression (LASSO regularization) and the random forest model, respectively, in order to avoid overfitting of our model, prior to its testing in an independent cohort. The index derived from our machine learning model was shown to have high prognostic significance and was the only significant feature to add value to a Cox regression model (Forward stepwise method), which included pT stage and the model’s individual components when using 5-year follow-up. The prognostic value of the index’s ability to identify a patient subpopulation with very good prognosis was then validated in an independent and international stage II CRC cohort without the need of any amendment of the thresholds of the image analysis or those used during the statistical analysis. To ensure that adjuvant treatment did not adversely affect the prognostic index, we excluded these patients from the training cohort as well as the validation cohort. Upon exclusion, the prognostic significance of the index did not change. This multi-parameter prognostic index demonstrates the importance of evaluating various TME components and their interactions, to better understand the complex factors that affect tumor progression. The translation of such indexes into a clinical setting, would aid in the identification of patients who may not require adjuvant treatment, which would correspondingly have a positive impact on the patient’s quality of life.

A key strength of this study was the use of automated image analysis for the classification and quantification of all cells and features reported. This therefore negates any potential inter and intraobserver variability^39^ and enables the standardization and reproducibility of the results. However, due to the heterogeneity across large patient populations, there exists variation in the intensity of signal to noise in the immunofluorescence intensity. To ensure artefact is not quantified as false positives, stringent thresholding was applied. This, however, can result in some positive-cell intensity lying below the set thresholds^40^. Despite this potential limitation, the results shown in this study stood up to scrutiny as all thresholds were kept constant across all images from the training and the validation cohort, which included patients from international institutes. We therefore demonstrate evidence that such methodologies can be widely applicable to the classification of cellular subtypes within the TME.

In this study, we first apply multiplex immunofluorescence on two sequential slides, which allows the quantification of the density and colocalization of cellular markers within distinct regions of the TME (IM, CT, IMCT). Second, using HALO^®^ image analysis software we automatically coregistered the serial sections, which offered the capability of studying the spatial interactions between multiple features within the TME. Finally, through a machine learning approach we demonstrate and validate a prognostic model, which could provide clinicians support in identifying patients with good prognosis, who would not need detailed follow-up or toxic, invasive, and expensive, further treatment. This would therefore result in an increase in patient quality of life and a decrease in costs to healthcare providers. Taken together, the findings of this study highlight the prognostic importance of capturing multiple distinct and interacting TME components in an objective and accurate way by the use of automated image analysis and machine learning.

## Methods

### Ethics statement

All experiments were conducted in accordance with the Declaration of Helsinki. The study was approved by the NHS Lothian NRS BioResourse and the ethical approval (13/ES/0126) was granted by East of Scotland Research Ethics Service as well as by the Ethics Committee of the National Defense Medical College (approval ref. No2992). Further ethical clearance was not required as the acquired data were anonymized.

### Patient cohort

We studied tumor specimens from 230 patients diagnosed with stage II CRC. One hundred and sixty-nine patients underwent surgical resection over the period 2002–2004 in Edinburgh, UK hospitals. Sixty-one patients underwent surgical resection over the years 2006–2011 at the National Defense Medical College Hospital, Japan. Sequentially selected specimens of patients from Edinburgh, between the years 2002–2003, were used as a training cohort (*n* = 113). All available patient specimens from Edinburgh treated within the year 2004 and the specimens from the Japanese cohort, were used as a validation cohort (*n* = 117). For each patient sample, all patient blocks were evaluated by the analysis of the corresponding hematoxylin and eosin stained tissue sections. A single tumor block containing the deepest tumor invasion was then selected for this study. Clinicopathological data, such as gender, age and TNM staging, were taken from the original pathology report. All clinicopathological data reported for both cohorts were compliant withTNM5 guidelines. Patient follow-up was up to 11.8 years for the Edinburgh patients in the training cohort, 10.2 years for the Edinburgh patients in the validation cohort and 8.6 years for the Japanese cohort. To ensure homogeneity between the Edinburgh and Japanese patients within the validation cohort, patients were censored at 8.6 years.

### Immunofluorescence and image capture

Antibody and immunofluorescence optimization was performed as previously described^14^. Briefly, brightfield uniplex immunohistochemistry was performed on 3-μm thick normal tonsil tissue sections to assess CD3 (rabbit polyclonal anti-human CD3, A045201-2, Dako), CD8 (mouse monoclonal anti-human CD8, M7103, Dako), CD68 (rabbit monoclonal anti-human CD68, 76437, Cell Signalling Technology), and CD163 (mouse monoclonal anti-human CD163, MRQ-26, Cell Marque) antibodies. A CRC tissue microarray was used for the assessment of PCK (mouse monoclonal anti-human cytokeratin, M3515012, Dako) primary antibody. Once antibody specificity was confirmed, uniplex immunofluorescence was used to optimize the primary and secondary antibodies as well as the associated fluorophores to be used for the visualization of each antibody. Multiplex immunofluorescence was then performed on two sequential 3-μm thick CRC tissue sections from the patient samples in each cohort. The first tissue section was labelled for CD3, CD8, PCK primary antibodies at 1:400, 1:200, and 1:100 dilutions, respectively, whereas the second tissue section was labelled for CD68, CD163, and PCK at 1:3000, 1:3000, and 1:100 dilutions, respectively. Hoechst (H3570, Thermo Fisher Scientific) was used for counterstaining both sections at a 1:20 dilution in deionized water. Tyramide signal amplification (TSA) FITC fluorescence kit (NEL741B001KT, PerkinElmer) was used for the visualization of CD3 and CD68 antibodies. TSA Cyanine 5 (Cy5) fluorescence kit (NEL745B001KT, PerkinElmer) was used for the CD8 and CD163 antibodies’ visualization. Anti-mouse Alexafluor 555 secondary antibody (A21422, Thermo Fisher Scientific) was used for PCK detection. Slides were mounted with ProLong Gold Antifade Reagent (P36930, Thermo Fisher Scientific). Immunofluorescence labelled slides were digitized using a Zeiss Axioscan.Z1 whole-slide scanner (Zeiss) through a 20× objective. Exposure times were as follows: FITC 25 ms for CD3 and 2 ms for CD68, Cy5 8 ms for CD8 and 10 ms for CD163, Cy3 120 ms for PCK of first slide and 15 ms for the sequential slide, Hoechst 12 ms for first slide and 1 ms for the sequential slide. These settings were kept constant across all patient cohorts.

### Image analysis

HALO® image analysis software (version 2.3.2089.34, IndicaLabs, Inc.) was used for whole-slide image analysis.

Quantification of the tumor infiltrating lymphocytes and TBs was performed as previously described^14^. Briefly, CD3^+^ and CD8^+^ lymphocytes were quantified using the High-Plex FL module within the IM (an area of 500-µm in and out of the invasive front), CT (the remainder tumor area), and both of these areas combined (IMCT) (Fig. 2a). Cell classification was based on dye intensity thresholds (CD3: FITC and CD8: Cy5) within segmented nuclei (dye-nucleus-positive threshold: FITC = 0.15, Cy5 = 0.132), the cytoplasm (cytoplasm-positive threshold: FITC = 0.5, Cy5 = 0.075), and the membrane (membrane-positive threshold: FITC = 0.5, Cy5 = 0.075). These thresholds were set identically for all patient samples. TBs were quantified in the TBROI (1000-μm border inward from the invasive front; Fig. 2a). Using the Area Quantification FL v1.2 module, the overall slide PCK intensity was measured and patients were dichotomized into low and high PCK categories (threshold = 2.16 × 10^−2^). Two image-based random forest classifiers; one for low PCK intensity images and one for the high PCK intensity images were used for the accurate tumor to stroma segmentation. Within the classified tumor regions, nuclei were segmented using a High-Plex FL module and tumor clusters of up to four cells in size were classified as TBs (Fig. 2b). Total TB number as well as TB and lymphocytic cell densities were exported from HALO^®^ image analysis software.

CD68^+^, CD163^+^, and CD68^+^CD163^-^ macrophages were quantified within the IM, CT, and IMCT (Fig. 2a). The High-Plex FL module was used for the classification of CD68^+^ (FITC), CD163^+^ (Cy5), and CD68^+^CD163^-^ cells, based on dye-nucleus-positive threshold (FITC = 0.200, Cy5 = 0.132), cytoplasm-positive threshold (FITC = 0.500, Cy5 = 0.075), and membrane-positive threshold (FITC = 0.500, Cy5 = 0.075; Fig. 2c). Cell densities within the two regions of interest were exported from HALO^®^ software.

Two images from the serial sections (1st with CD3, CD8, PCK, Hoechst and 2nd with CD68, CD163, PCK, Hoechst) were automatically coregistered within HALO® software (Fig. 2d).

After coregistration, the spatial coordinates of the lymphocytes, TBs and macrophages were imported into a two-dimensional spatial plot. Within this plot the spatial relationships between macrophages and TBs, as well as macrophages and lymphocytes, were assessed. To do so, CD68^+^, CD163^+^, and CD68^+^CD163^-^ cells that were present within 0–50-μm and 0–100-μm radii from the TBs, CD3^+^, or CD8^+^ cells were quantified.

### Statistical analysis

The automatic quantification of the numbers, densities, and spatial relationships of CD3^+^, CD8^+^, CD68^+^, CD163^+^, CD68^+^CD163^-^ cells, and TBs resulted in 69 image-based features. These features together with the data from the clinicopathological report, were imported into RStudio 1.1.419^41^ running R 3.4.3^42^. The relationship between lymphocytes, macrophage density and TB number was assessed on the training cohort using the Spearman correlation coefficients (r). Univariate Cox regression was applied to the clinicopathological data to assess their prognostic significance. *P*-values of less than 0.05 were considered statistically significant. A Cox regression with LASSO regularization was applied to all image analysis features as well as the data from the clinicopathological report. This was performed to reduce the feature dimensionality of the dataset by identifying the most prognostically significant features. During this process, a 10-fold cross validation was performed to avoid overfitting. The independent prognostic significance of the selected features was assessed using a multivariate Cox regression in SPSS^43^. The data from each selected significant feature were then input into a random forest (*n* = 500) decision tree model. The features were then ranked according to their mean decrease Gini coefficient^44^. Features with a mean decrease Gini of greater than 3 were then assessed for their correlation to each other. For any highly correlated features, only the one with the highest Gini coefficient was selected for further analysis. Optimal cutoff points for the selected features were calculated based on disease-specific survival using the survminer package^45^. Iterative combinations of the selected features were tested and the model with highest prognostic significance was chosen. Univariate Cox regression and KM survival analysis were performed to assess the SIOI and compared it with its individual components and pT stage. Multivariate Cox regression with forward selection was also applied to compare the prognostic values of the SIOI, its components and pT stage. We then assessed the prognostic significance of our model on an independent validation cohort by directly applying the cutoff points obtained from the training cohort. Disease-specific survival, defined as the period of time (in months) from surgical resection to the date of death from CRC or the last follow-up date for patients still alive, was used for all our survival analysis.

### Reporting summary

Further information on experimental design is available in the Nature Research Reporting Summary linked to this article.

## Supplementary information

Supplemental Material

Reporting Summary

## Data Availability

The data that support the findings of this study are available from the corresponding author upon reasonable request.

## Code availability

Image analysis was performed using commercially available HALO^®^ image analysis software. The specific modules used for the classification and quantification of all image analysis features are described in the “Methods” section. All data analysis was performed using RStudio version 1.1.419^41^ running R version 3.4.3^42^ and SPSS version 24^43^ software. The data analysis code is available from the corresponding author upon request.
