# Supplementary material for: Spatial immune profiling of the colorectal tumor microenvironment predicts good outcome in stage II patients
Source: NPJ Digit Med. 2020 May 15;3:71. doi: 10.1038/s41746-020-0275-x (PMC7229187; doi:10.1038/s41746-020-0275-x)
Supplement: Supplementary file 1 — Supplemental Material [file 41746_2020_275_MOESM1_ESM.pdf]

Supplementary Materials

Supplementary Table 1. *P* values of association between macrophage and lymphocytic infiltration and tumor buds. Abbreviations: IM, invasive margin; CT, tumor core; IMCT, invasive margin and tumor core; TB, Tumor bud.

Supplementary Table 2. Cut-off values for Spatial Immuno-Oncology (SIOI) components. Abbreviations: IMCT, Invasive margin and tumor core; TB, Tumor bud; CT, tumor core.

Supplementary Table 3. Features selected by Cox regression with LASSO regularization entered into a multivariate Cox regression. Independent feature significance is shown. Abbreviations: HR, hazard ratio; CI, confidence interval; IMCT, invasive margin and tumor core; TB, tumor bud; CT, tumor core; EMLVI, extramural lymphovascular invasion.

Supplementary Table 4. Features entered into the multivariate forward stepwise cox regression for 11.5-year survival.

Supplementary Table 5. Features entered into the multivariate forward stepwise cox regression for 5-year survival.

Supplementary Figure 1. Kaplan Meier survival analysis for 2-tier Spatial Immuno-Oncology Index (SIOI) for training cohort (11.8 and 5-year follow–up) and validation cohort (8.6 and 5-year follow up).

Supplementary Figure 2. Kaplan Meier survival analysis for Spatial Immuno-Oncology Index (SIOI) for a divided validation cohort using full follow-up.

Feature	CD3.IM	CD3.CT	CD3.IMCT	CD8.IM	CD8.CT	CD8.IMCT	TB.Number	CD68.IM	CD68.CT	CD68.IMCT	CD163.IM	CD163.CT	CD163.IMCT	CD68^{+}CD163^{-}.IM	CD68^{+}CD163^{-}.CT	CD68^{+}CD163^{-}.IMCT
**CD3.IM**	NA	0.000	0.000	0.000	0.000	0.000	0.024	0.021	0.000	0.018	0.036	0.285	0.113	0.001	0.002	0.002
**CD3.CT**	0.000	NA	0.000	0.051	0.081	0.053	0.800	0.667	0.105	0.482	0.675	0.889	0.789	0.113	0.154	0.167
**CD3.IMCT**	0.000	0.000	NA	0.000	0.001	0.000	0.415	0.342	0.023	0.266	0.312	0.789	0.551	0.014	0.017	0.023
**CD8.IM**	0.000	0.051	0.000	NA	0.000	0.000	0.058	0.202	0.013	0.072	0.004	0.010	0.010	0.108	0.063	0.079
**CD8.CT**	0.000	0.081	0.001	0.000	NA	0.000	0.100	0.899	0.168	0.613	0.003	0.002	0.002	0.103	0.125	0.099
**CD8.IMCT**	0.000	0.053	0.000	0.000	0.000	NA	0.059	0.599	0.052	0.257	0.002	0.002	0.003	0.084	0.073	0.069
**TB.Number**	0.024	0.800	0.415	0.058	0.100	0.059	NA	0.524	0.021	0.088	0.165	0.097	0.171	0.567	0.014	0.100
**CD68.IM**	0.021	0.667	0.342	0.202	0.899	0.599	0.524	NA	0.000	0.000	0.005	0.967	0.227	0.000	0.000	0.000
**CD68.CT**	0.000	0.105	0.023	0.013	0.168	0.052	0.021	0.000	NA	0.000	0.184	0.171	0.525	0.000	0.000	0.000
**CD68.IMCT**	0.018	0.482	0.266	0.072	0.613	0.257	0.088	0.000	0.000	NA	0.035	0.631	0.433	0.000	0.000	0.000
**CD163.IM**	0.036	0.675	0.312	0.004	0.003	0.002	0.165	0.005	0.184	0.035	NA	0.000	0.000	0.022	0.087	0.072
**CD163.CT**	0.285	0.889	0.789	0.010	0.002	0.002	0.097	0.967	0.171	0.631	0.000	NA	0.000	0.050	0.086	0.059
**CD163.IMCT**	0.113	0.789	0.551	0.010	0.002	0.003	0.171	0.227	0.525	0.433	0.000	0.000	NA	0.007	0.024	0.014
**CD68^{+}CD163^{-}.IM**	0.001	0.113	0.014	0.108	0.103	0.084	0.567	0.000	0.000	0.000	0.022	0.050	0.007	NA	0.000	0.000
**CD68^{+}CD163^{-}.CT**	0.002	0.154	0.017	0.063	0.125	0.073	0.014	0.000	0.000	0.000	0.087	0.086	0.024	0.000	NA	0.000
**CD68^{+}CD163^{-}.IMCT**	0.002	0.167	0.023	0.079	0.099	0.069	0.100	0.000	0.000	0.000	0.072	0.059	0.014	0.000	0.000	NA

Supplementary Table 1. P values of association between macrophage and lymphocytic infiltration and tumor buds. Abbreviations: IM, invasive margin; CT, tumor core; IMCT, invasive margin and tumor core; TB, Tumor bud.

Feature	Cut-off value
CD3^{+} in IMCT	389.6
CD3^{+} CD8^{+} 0-50 TB	4.1
CD68^{+}/CD163^{+} ratio in the CT	1.0955

Supplementary Table 2. Cut-off values for Spatial Immuno-Oncology Index (SIOI) components. Abbreviations: IMCT, Invasive margin and tumor core; TB, Tumor bud; CT, tumor core.

Features	*P* value	HR	95.0% CI	
			Lower	Upper
**$CD3^+$ in IMCT**	0.794	1.000	.997	1.003
**$CD3^+CD8^+$ within 0-50μm of TB**	**0.017**	**0.745**	**.585**	**.948**
**$CD68^+/CD163^+$ in CT**	0.417	0.722	.329	1.585
**$CD68^+CD163^-$ in CT**	**0.014**	**1.005**	**1.001**	**1.010**
**$CD8^+$ in CT**	0.066	0.997	.993	1.000
**TB Number**	0.558	1.000	1.000	1.001
**$CD163^+$ within 0-50 of $CD8^+$**	0.808	1.000	.997	1.002
**pT**	**0.027**	**5.046**	**1.204**	**21.146**
**EMLVI**	0.128	0.328	.078	1.378
**Age**	**0.005**	**4.321**	**1.542**	**12.107**
**Differentiation**	**0.005**	**4.892**	**1.607**	**14.893**

Supplementary Table 3. Features selected by Cox regression with LASSO regularization entered into a multivariate Cox regression. Independent feature significance is shown. Abbreviations: HR, hazard ratio; CI, confidence interval; IMCT, Invasive margin and tumor core; TB, Tumor bud; CT, tumor core; EMLVI, extramural lymphovascular invasion.

Variables in the equation	Multivariate Cox Regression Model			
	**HR**	**95% CI**		**P Value**
		**Lower**	**Upper**	
SIOI	6.119	2.661	14.069	< 0.001
pT stage	3.531	1.260	9.893	0.016
**Variables not in equation**				
$CD3^+$ in IMCT				NS
$CD3^+$ $CD8^+$ 0-50 TB				NS
$CD68^+/CD163^+$ in CT				NS

Supplementary Table 4. Features entered into the multivariate forward stepwise cox regression for 11.5-year survival. Features which add significance (on the top half of table) and features which do not add significance (on the bottom half of the table) to a model to predict disease specific death. Abbreviations: HR, hazard ratio; CI, confidence interval; SIOI, Spatial Immuno-Oncology Index; IMCT, Invasive margin and tumor core; TB, Tumor bud; NS, Non-significant.

Variables in the equation	Multivariate Cox Regression Model			
	HR	95% CI		P Value
		Lower	Upper	
SIOI	5.508	1.910	15.884	0.002
**Variables not in equation**				
pT stage				NS
$CD3^{+}$ in IMCT				NS
$CD3^{+}$ $CD8^{+}$ 0-50 TB				NS
$CD68^{+}/CD163^{+}$ in CT				NS

Supplementary Table 5. Features entered into the multivariate forward stepwise cox regression for 5-year survival. Features which add significance (on the top half of table) and features which do not add significance (on the bottom half of the table) to a model to predict disease specific death. Abbreviations: HR, hazard ratio; CI, confidence interval; SIOI, Spatial Immuno-Oncology Index; IMCT, Invasive margin and tumor core; TB, Tumor bud; NS, Non-significant.

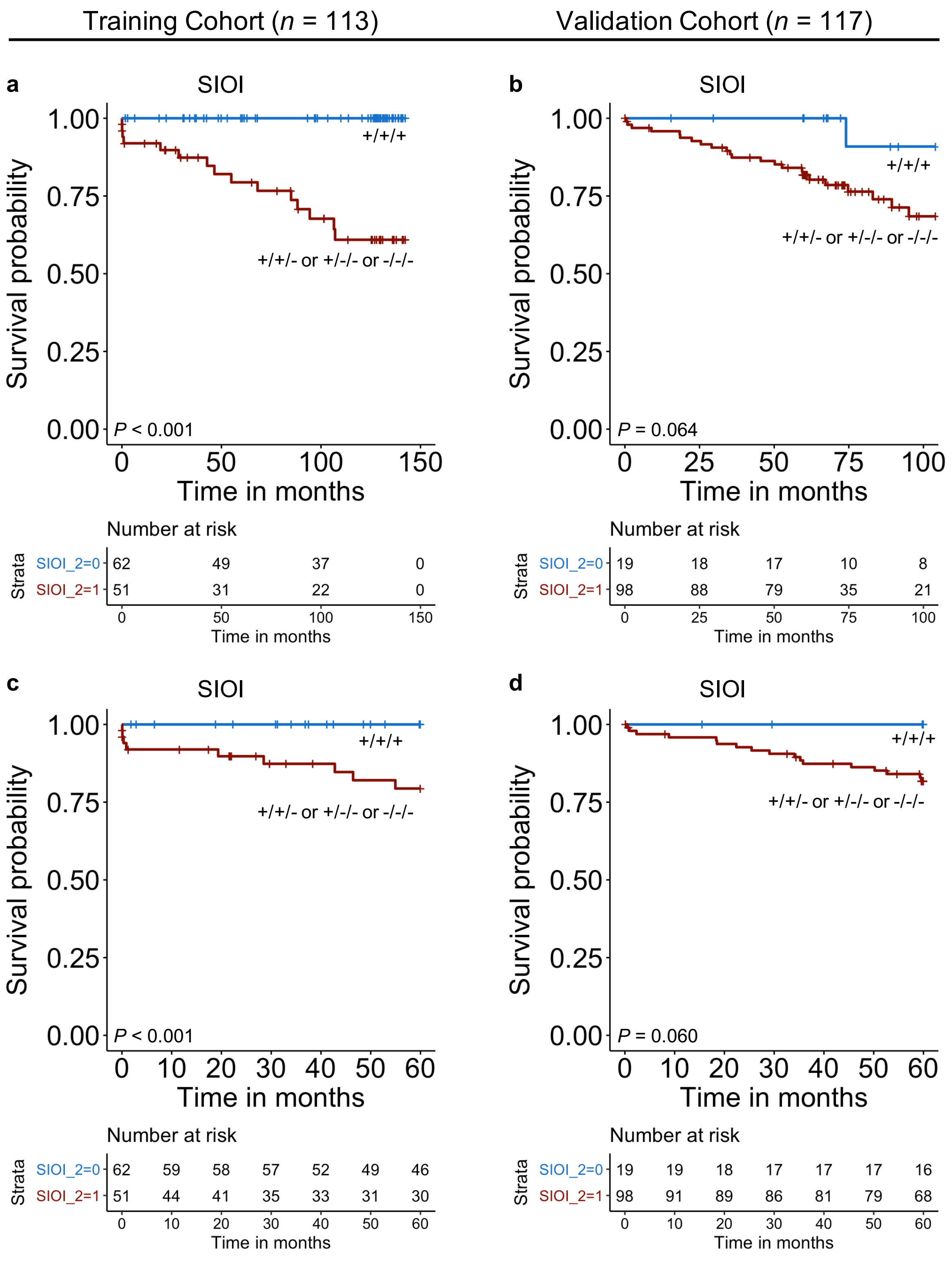

Supplementary Figure 1. Kaplan Meier survival analysis for 2-tier Spatial Immuno-Oncology Index (SIOI) for training cohort (11.8 and 5 year follow–up) and validation cohort (8.6 and 5 year follow

up). (a to b) SIOI for cohorts using full follow-up. (c to d) SIOI for cohorts using 5-year follow-up. "+/+/+" category represents the group of patients who have CD3+ density in the invasive margin and tumor core above the cut-off point (389.6 cells/mm2), mean CD3+CD8+ number within 0-50µm of TB above the cut-off point (4.1) and CD68+ /CD163+ ratio below the cut-off point (1.096); "+/+/-, +/-/-, -/-/-" group represents patients who have only 2, 1 or none of these features respectively.

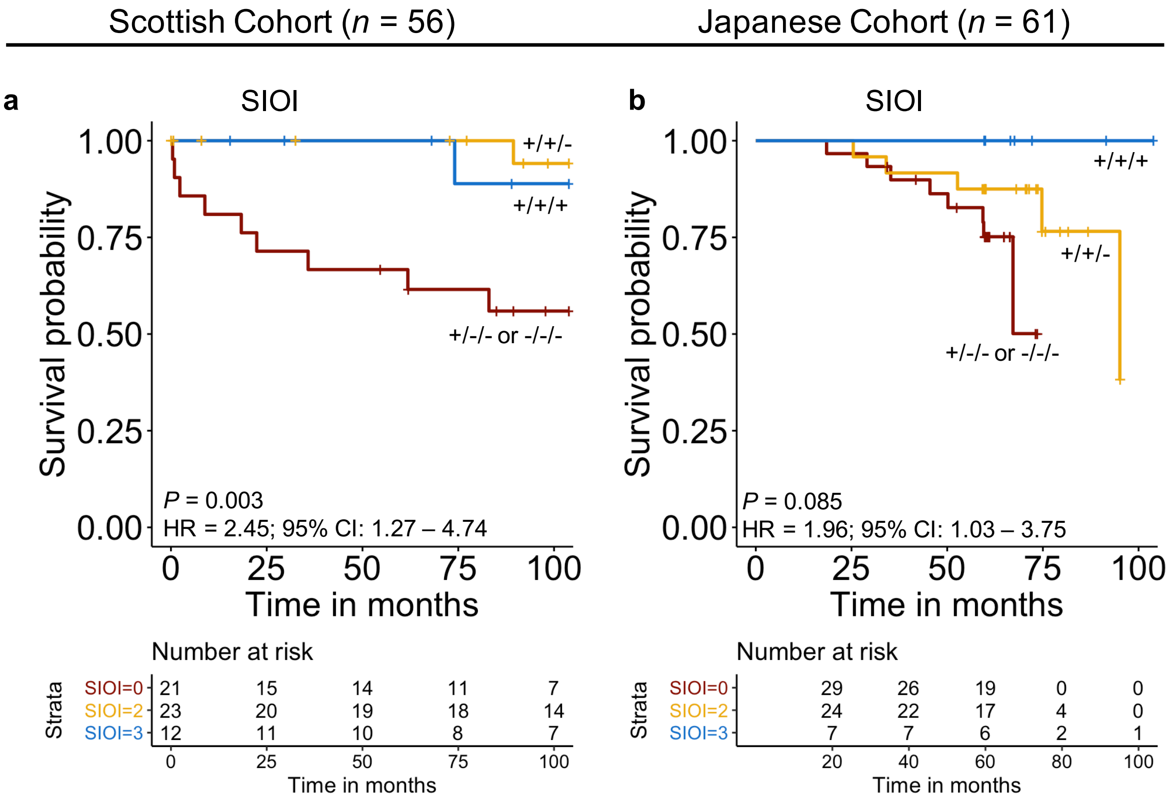

Supplementary Figure 2. Kaplan Meier survival analysis for Spatial Immuno-Oncology Index (SIOI) for a divided validation cohort using full follow-up. (a) SIOI for Scottish cohort. (b) SIOI for Japanese cohort. "+/+/+" represents the group of patients who have CD3+ density in the invasive margin and tumor core above the cut-off point (389.6 cells/mm2), mean CD3+CD8+ number within 0-50µm of TB above the cut-off point (4.1) and CD68+ /CD163+ ratio below the cut-off point (1.096); "+/+/-" group represents patients who are positive for only 2 of these features and "+/-/- or -/-/-" represents the group of patients who have only 1 or none of the above features. Hazard ratios (HR) are calculated using univariate Cox regression.
